# Involvement of the cerebellum in the regulation of executive functions in children—Preliminary analysis based on a neuropsychological study of children after cerebellar tumour surgery

**DOI:** 10.3389/fpsyg.2022.961577

**Published:** 2022-10-06

**Authors:** Anna Starowicz-Filip, Barbara Bętkowska-Korpała, Tetiana Yablonska, Stanisław Kwiatkowski, Olga Milczarek, Łukasz Klasa, Adrian Andrzej Chrobak

**Affiliations:** ^1^Department of Medical Psychology, Faculty of Medicine, Jagiellonian University Medical College, Kraków, Poland; ^2^Department of Developmental Psychology, Faculty of Psychology, Taras Shevchenko National University of Kyiv, Kyiv, Ukraine; ^3^Department of Pediatric Neurosurgery, Faculty of Medicine, Jagiellonian University Medical College, Kraków, Poland; ^4^Department of Adult Psychiatry, Faculty of Medicine, Jagiellonian University Medical College, Kraków, Poland

**Keywords:** cerebellum, executive functions, IDS-2 Scale, CCAS syndrome, children

## Abstract

**Aim:**

Preliminary assessment of executive functions in children with cerebellar lesions, description of their emotional-social functioning and selection of sensitive neuropsychological tools to detect the cerebellar cognitive affective syndrome (CCAS).

**Materials and methods:**

The study group consisted of 10 children after cerebellar tumour surgery. The control group consisted of 10 healthy children, matched for age and sex: The IDS-2 executive functions battery, the Conners 3 ADHD questionnaire, the Autism Spectrum Rating Scales (ASRS) and the International Cooperative Ataxia Rating Scale (ICARS) were used.

**Results:**

Statistical analysis showed statistically significant differences between the experimental and control groups in terms of two dimensions of executive functioning. Children from experimental group was characterised by worse planning and divided attention than healthy controls. Moreover children with cerebellar lesions were characterised by significantly higher levels of some behaviours similar to that observed in autism spectrum disorders, namely difficulties in social relationships, self-regulation of emotions, attention, and greater behavioural rigidity. Test power analysis and estimation of the effect size by the Cohen’s *d* coefficient indicated that with a slight increase in the size of the experimental group, the probability of detecting statistically significant difference in the executive functions total measure score as well as in several ASRS subscales increased, but not in Conners 3 subscales.

**Conclusions:**

Cerebellar damage may pose a risk for dysexecutive syndrome and social-emotional problems in children. The IDS-2 executive functions battery and the ASRS test are sufficiently sensitive tools to assess elements of the CCAS in children.

## Introduction

The publication by Schmahmann and Sherman (1998) on the cerebellar cognitive affective syndrome led to a marked increase in researcher interest in the involvement of the cerebellum in the course of higher mental functioning. Research in this area points to its association with the performance of such cognitive domains as language function and verbal fluency (Arasanz et al., 2012), attention (Steinlin et al., 2003; Beebe et al., 2005), visuospatial functions (Starowicz-Flip et al., 2015), working memory (Andreasen et al., 1999; Exner et al., 2004; Fliessbach et al., 2007), and executive functions (Levisohn et al., 2000). The role of the vermis in the regulation of emotional states has also been highlighted (Levisohn et al., 2000; Steinlin et al., 2003). In explaining the observed correlations, researchers refer to neuroanatomical data, pointing to the existence of significant connections between the cerebellum and association regions of the cerebral cortex, especially with the prefrontal region of the CNS (Ramnani, 2006; Grimaldi and Manto, 2011), which would explain the observed pseudo-frontal character of cognitive deficits affecting patients with cerebellar damage (Starowicz-Filip et al., 2020).

Studies in the paediatric population, although far less numerous, indicate the presence of the cerebellar cognitive affective syndrome (CCAS) also in children. Cognitive impairment is marked in post-surgery examinations of children with tumours of this structure (Levisohn et al., 2000; Beebe et al., 2005; Konczak et al., 2005; Moore et al., 2017), both those with a high degree of malignancy (medulloblastoma) and those of a low grade pathology (pilocytic astrocytoma) (Catsman-Berrevoets and Aarsen, 2010).

There are also isolated reports of lower intellectual performance in children with posterior fossa cysts, although in this area the results are far less consistent (Cuny et al., 2017; Guell et al., 2020).

Studies show that about 66% of children after surgery for cerebellar tumours have school difficulties, only 40% of children after surgery for medulloblastoma reach an adequate level of education five years after the operation and 26% of them require special education (Hoppe-Hirsch et al., 1995). The school difficulties in these children cannot be solely attributed to surgery. In patients after treatment of medulloblastoma, sequalae of radiation therapy plays a large role in reducing IQ, due to the impairment of processing speed which has a profound influence on diminishing performance of working memory and attention (King et al., 2019).

Parents of children who have undergone surgery for cerebellar tumours describe attention deficits, difficulty in concentrating in class, inability to plan homework, and chaotic way of learning. There are also observations of emotional difficulties in these children, consisting of increased impulsivity, irritability, impatience, agitation and anxiety. On the other extreme of the reported changes remains increased psychomotor retardation, decreased initiative, and difficulty in engaging in peer relationships. Parents of children after surgery for cerebellar tumours listed hyperactivity and frequent tantrums, meticulousness, explosive behaviour, crying days, shutting down in new environment, shyness, quietness, learning problems, memory and other difficulties.

The above description of difficulties may correspond to the pattern of executive function disorders understood as a series of higher mental processes, such as planning, problem solving, decision making, working memory, abstracting, cognitive flexibility, inhibitory control, and adaptation to new circumstances and demands (Karatekin et al., 2000).

There is a relatively large number of studies highlighting the role of the cerebellum in the regulation of executive functions, but they mainly involve adults (Fiez et al., 1992; Grafman et al., 1992; Bellebaum and Daum, 2007). Some of them suggest that the cerebellar cortex may not be critical for executive functions instead there is a claim that the cerebellum has a supportive role characterised by its computing of the motor requirements when executive functions processing is required (Beuriat et al., 2020). There is far less research in children concerning the role of the cerebellar lesions in executive functioning (Steinlin et al., 2003; Koustenis et al., 2016; Albazron et al., 2019). As shown by the Beuriat and colleagues (Beuriat et al., 2022), the role of the cerebellum in the regulation of executive, emotional and social factors is changing across lifespan. As a result of anatomical and functional changes, neuroimaging and clinical data indicate that the importance of the role of the cerebellum in human executive functions-related networks shifts from being crucial in newborns and young children to being only supportive in adult life.

Executive function performance is conditioned by the emotional state (Ferrier et al., 2014). On the other hand, damage to the cerebellum contributes to the occurrence of difficulties of emotional nature (Adamaszek et al., 2017), which may indirectly determine the subsequent executive performance of patients. It is worth mentioning that changes in the cerebellar structure are documented in children with ADHD (Castellanos et al., 2002), or autism (Townsend et al., 2001).

The question arises, therefore, as to how presence of a lesion in the cerebellum affects executive functions, emotional processes (including regulation of emotions and behaviour) and social functioning of a child? Knowledge of the specific executive and emotional difficulties of children with cerebellar damage would allow not only to predict their further coping at school, but also to implement appropriate neuropsychological rehabilitation programmes to compensate for the deficits.

The aim of our study was to preliminary assess the profile of executive functions in children who underwent surgery for low-grade cerebellar tumours. The second goal was also to describe the emotional and social functioning, which may subsequently determine the executive performance of the patients studied. The nature of the study was exploratory and preliminary, with the additional aim of identifying neuropsychological tools sensitive enough to detect changes in the executive and behavioural functioning of these children. This is particularly important because, as Leiner et al. (1991) write, the lack of intellectual disturbances described in some studies in cerebellar patients (Daum and Ackermann, 1997) is due to the fact that they may be so selective and subtle that they require the selection of targetted and sensitive neuropsychological tools. Indeed, the cerebellum itself is seen as ‘a modulator rather than a generator of cognitive functioning’ (Schmahmann et al., 2019).

The following research questions were posed:

1. Do children with cerebellar lesions have impaired performance of the executive functions, i.e., planning ability, impulsivity control, automatic response inhibition, and divided attention?
2. What is the profile of executive dysfunctions in these children; which functions are preserved and which are impaired?
3. Do children with cerebellar lesions manifest emotional and social difficulties and to what extent do these affect the efficiency of executive functions?

## Materials and methods

Ten children with cerebellar lesions after the surgery for low-grade cerebellar tumour, WHO grade I and II, histopathological type of tumour: astrocytoma, participated in the study within the experimental group. The study group consisted of patients operated on in the Department of Neurosurgery of the Children’s University Hospital in Kraków, attending the neuropsychological examination, treated on an outpatient basis. The mean time since surgery was 18.8 months (range 2 month–5 years). The mean age during the neuropsychological examination was 10.4 years (range 7–15 years). The study group consisted of 5 girls and 5 boys. No postoperative chemo- or radiotherapy was used in these children. No hydrocephalus requiring shunting system implantation was found in this group. Although brain tumours located in the posterior fossa in children represent the highest incidence (Steinlin et al., 2003), cerebellar tumours are usually the most malignant. Benign cerebellar tumours are relatively rarer, therefore the group described in the context of the initial report is small. The exclusion criteria were presence of extracerebellar CNS damage, premorbid intellectual disability and premorbid serious behavioural and emotional problems, typical for ADHD or Autism Spectrum Disorder ASD and cerebellar mutism syndrome. Since premorbid testing was not available for the participants after cerebellar tumour surgery, the information of the lack of neurological, cognitive dysfunctions and serious emotional problems including ASD was based on parents’ opinions. This solution was also used previously in other research (Levisohn et al., 2000).

The control group consisted of 10 children without organic CNS damage, matched for age during the neuropsychological exam (mean age 11,1, range 7–16) and gender, who were hospitalised at the neurosurgery department and orthopaedic outpatient clinic due to non-cerebral dysfunctions (spinal disorders, spinal cord injury, spinal cord tumour or scoliosis).

The following research tools were used in the study:

The IDS-2 battery of executive functions from the Intelligence and Development Scale for Children and Adolescents (Polish adaptation by Jaworowska et al., 2018). This tool consists of four subscales:

– Listing words: Tasks for younger children (5–9 years), i.e., listing within 90 s the names of animals, followed by things to eat. Tasks for older children (10–20 years), i.e., naming animals within 90 s, alternating between vehicles and vegetables or fruit, letter O words alternating with words beginning with the letters S and W. The tasks measure semantic and phonological verbal fluency, cognitive flexibility, the ability to retrieve words quickly from long-term lexical memory and to keep them in short-term memory so as not to repeat them, and the use of strategies to organise the word recall process.
– Divided attention: the subject is asked to quickly search for and cross out visual stimuli (drawings of parrots) with specific features, while at the same time listing words belonging to a specific category (animal names). The test examines the ability to divide attention resources into two simultaneous sentences, but also the speed of processing visual information, working memory, selectivity, inhibition of reactions to distractors.
– Animal colours: the essence of the task consists in giving the actual, and at the same time inconsistent with the image seen, colours of the animals depicted in the drawings. The main object of measurement here is the ability to overcome interference, and inhibit the dominant response, which consists in naming the actual colours seen (Stroop paradigm).
– Drawing routes: consists in following drawn routes with a pen, which in more difficult tasks resemble mazes, without returning to the sections already covered. This requires advance planning of the routes. In addition to the ability to plan, the test also involves visuospatial skills and the ability to inhibit and restrain automatic reactions and impulsivity, the tendency to act quickly without thinking.
– Executive total score is a sum of scores of four subscales, mentioned above.

The IDS-2 Screening IQ test, from the Intelligence and Development Scale for Children and Adolescents, consisted of the sum of results of two IDS-2 subscales: Matrices and Categories (Polish adaptation by Jaworowska et al., 2018).

The International Cooperative Ataxia Rating Scale (ICARS) (Trouillas et al., 1997) consists of 19 items divided into 4 groups of ataxia symptoms: postural and gait disturbances (7 items, 34 points maximum), limb ataxia (7 items, 52 points maximum), dysarthria (2 items, 8 points), and oculomotor disorders (3 items, 6 points). The maximum score for the whole test is 100 points. The higher the score, the greater the severity of cerebellar ataxia symptoms. This scale was previously used in children with cerebellar damage (Kieffer et al., 2012).

Conners 3—a set of questionnaires for the diagnosis of behaviour typical for ADHD and co-occurring disorders, third edition in Polish adaptation (Wujcik and Wrocławska-Warchala, 2018). Parent version.

Parents provide answers regarding the extent to which the behaviour described in the questionnaire has been observed in their children in various everyday life situations. The questionnaire contains scales directly based on the DSM-5 diagnostic criteria for ADHD and the most common co-occurring disorders such as oppositional defiant disorder. From the perspective of our study, however, more important are the so-called content scales, which also describe difficulties in executive functions, learning, and behaviour.

Interpretation of content scales:

– inattention (attention deficit disorder, errors resulting from carelessness, difficulties in starting and finishing tasks), hyperactivity/impulsivity, learning problems, executive functions, disobedience/aggression, peer relations (ability to make friends, difficulties in being accepted in a group), family relations.

• The Autism Spectrum Rating Scales (ASRS) – a set of questionnaires in Polish adaptation (Wrocławska- Warchala and Wujcik, 2016). Parent version.

ASRS scales:

– social/communication (ability to engage in social contact, maintain relationships, appropriate use of verbal and non-verbal communication);
– unusual behaviours (difficulty tolerating changes in routine activities, stereotyped behaviour, sensory sensitivity); and
– self-regulation (deficits in attention, motor control and impulse control).

Treatment scales: peer socialisation, adult socialisation, social/emotional reciprocity, atypical language, stereotypy, behavioural rigidity, sensory sensitivity, attention/self-regulation.

The questionnaire contains scales directly based on the DSM-4 diagnostic criteria for ASD.

The last two questionnaires were treated as an attempt to capture and describe the behavioural-emotional difficulties reported by the parents of the operated children, marking them as a clear, qualitative post-operative change in behaviour in the context of the child’s pre-disease functioning. The description was not aimed at a diagnosis of ADHD or autism, but an attempt to capture some characteristic components, treated in this group as a behavioural-emotional symptom of cerebellar damage.

The detailed characteristics of the patients group along with the overall results obtained in the individual questionnaires are included in Table 1.

**TABLE 1:** Detailed characteristics of the patients with cerebellar lesion.

Patient	sex	age	Location of cerebellar tumour	Histopathology	Time since surgery	IDS-2 IQ screening results	Executive functions – general result	Conners 3 scores	ASRS general result	ICARS total score	Parents’ observations of changes in their child’s behaviour after surgery in contrast to the child typical functioning/data from interview
1	boy	12 years old	Left cerebellar hemisphere	Astrocytoma WHO II	2 years	106	12	Learning problems	42 average score	3	Hyperactivity Increased tic intensity Difficulty concentrating attention
2	girl	15 years old	Vermis and bilaterally medial parts of the cerebellar hemispheres	Astrocytoma WHO I/II	5 years	91	4	Inattention Learning problems Executive functioning Peer relations	75 very high score	12	Autistic behaviour Rigidity of rituals/compulsions Social withdrawal Inadequacy of emotional reactions
3	girl	7 years old	Right cerebellar hemisphere	Astrocytoma WHO I	6 months	121	9	None	55 average score	4	Parents reported no negative changes in emotions or cognitive processes
4	boy	7 years old	Right cerebellar hemisphere	Astrocytoma I	2 months	104	8	None	26 low score	5	Parents reported no negative changes in emotions or cognitive processes
5	girl	11 years old	Left cerebellar hemisphere and vermis	Astrocytoma	1 year	61	1	Inattention Executive functions	75 very high score	32	Impulsivity, irritability, tantrums, difficulty in controlling emotions, Difficulty in learning, concentration, difficulty in forming longer speech, speech limited to simple, short sentences
6	boy	8 years old	Right cerebellar hemisphere	Astrocytoma I/II	1 year	100	5	Inattention Hyperactivity Impulsivity	54 average score	0	Parents reported no emotional or behavioural difficulties
7	boy	12 years	Cerebellar vermis	Astrocytoma I/II	2 months	107	5	Inattention Hyperactivity/Impulsivity Learning problems Executive functioning Peer relations	81 very high score	2	The child has been hyperactive from an early stage after the surgery, difficulty concentrating attention and learning at school
8	girl	12 years	Vermis and right cerebellar hemisphere	Astrocytoma I/II	3 years	76	1	Inattention Executive functions	61 elevated score	29	Parents reported impulsiveness, low mental flexibility, difficulty in changing behaviours
9	boy	9 years	Vermis and both cerebellar hemispheres	Astrocytoma I/II	1 month	97	12	None	42 average score	12	The child spoke little, using short sentence, the speech was simplified, with decrease of the motivation to make some verbal descriptions, problems with concentration
10	girl	10 years	Left cerebellar hemisphere	Astrocytoma I	1 year	94	6	None	74 very high score	4	Difficulty in controlling emotions, social withdrawal

Executive function general results—the sum of sores in four executive functions subscales: listing words, animal colours, divided attention and drawing routs (scores range from 1 to 19, points between 7 and 13 are the average score, scores below 7 express the problems in executive functions.

ASRS questionnaire: T- scores, range between 10 and >90, the average range is usually between 40 and 60 T-scores.

Elevated results are over 60 T-score, hight scores over 65T-scores, very hight scores over 70 T-scores, the higher scores the higher intensification of autism like symptoms.

### Procedure

The neuropsychological examination of children was done by the clinical neuropsychologist and took about 45 min/1 h. In that time the parent of the child was filling the questionnaires: Conners-3 and ASRS. Parents of the patients were also asked about the changes of emotional and behavioural functioning of the children after the surgery in contrast to the typical child’s behaviour during its life. The children included in the study were assessed by a neurologist before the neuropsychological examination, according to their motor functions and neurological state. All participants were informed of the possibility of stopping the testing or taking a break if it became too demanding. All test instructions were carefully explained. The participants could ask any question during the testing.

## Results

The Mann–Whitney *U* Test analysis was performed to compare patients with lesions located in or around the cerebellum with a control group of healthy children in terms of both general intelligence, executive functions and emotional functioning. The results are presented in Table 2.

**TABLE 2:** Comparison of the study and control group in terms of intellectual and emotional variables (the statistically significant results are bolded).

Psychological tests	Patients Median (upper and lower quartile)	Controls Median (upper and lower quartile)	U value	*r* r = z/$\sqrt{n}$	*p*	Effect size Cohen’s *d* factor	Test power
**IDS-2**							
Matrices	9.00(6.50;11.25)	11.50(10.00;12.25)	22.50	0.467	**0.035**	1.11	0,75
Categories	11.00(8.50;12.00)	13.50(11.50;15.25)	16.50	0.568	**0.009**	1.33	0.87
IDS-2 IQ screening result	98.50 (87.25;106.25)	113.50(108.25;122.50)	13.00	0.626	**0.004**	1.36	0.87
**IDS-2 executive functioning**							
Listing words/Fluency	8.50(7.00;11.25)	10.00(7.00;11.25)	40.50	0.161	0.481	0.24	0.12
Divided attention	7.00(2.50;9.00)	10.00(7.75;11.50)	16.50	0.570	**0.009**	1.39	0.89
Animal colours	10.50(4.75;11.00)	10.00(9.00;13.50)	41.50	0.145	0.529	0.67	0.41
Drawing routes	5.00(4.00;8.50)	9.50(7.75;13.25)	23.00	0.462	**0.043**	1.01	0.68
Executive functioning - total	7.00(3.25;9.75)	9.50(7.75;13.00)	25.50	0.416	0.063	0.94	0.63
**Conners 3 questionnaire**							
Inattention	55.00(43.75; 76.00)	48.50(40.00;67.25)	41.50	0.144	0.529	0.36	0.19
Hyperactivity/Impulsivity	49.50(41.50;53.50)	44.50(40.00;62.25)	45.00	0.085	0.739	0.04	0.06
Learning problems	50.00(41.75; 60.00)	50.00(41.50;57.00)	45.00	0.084	0.739	0.26	0.13
Executive functioning	52.00(42.25;70.00)	46.00(40.75;61.50)	40.50	0.161	0.481	0.33	0.17
Disobedience/aggression	47.50(41.50;54.00)	45.50(40.75;56.50)	49.50	0.008	0.971	0.13	0.08
Peer relations	45.50(43.00;55.25)	42.50(40.75;47.75)	30.00	0.340	0.143	0.59	0.34
**ASRS questionnaire**							
Social relationships/communication	54.00(45.25;78.00)	24.00(21.00;36.75)	2.50	0.806	**0.001**	2.39	1.00
Unusual behaviours	46.50(30.75;53.00)	24.00(22.50;31.00)	16.00	0.577	**0.009**	1.36	0.89
Self-regulation	59.50(43.25;67.50)	30.00(25.00;48.50)	14.00	0.610	**0.005**	1.26	0.84
General result	54.50(39.75;74.25)	21.00(19.00;31.75)	8.00	0.712	**0.001**	1.99	0.99
ASRS DSM	50.00(35.00;71.00)	22.00(20.50;27.25)	3.00	0.798	**0.001**	2.24	1.00
Peer socialisation	52.50(35.50;75.00)	28.00(27.50;31.00)	14.00	0.618	**0.005**	1.57	0.95
Adult socialisation	49.00(37.75;57.25)	30.00(27.75;36.25)	13.50	0.806	**0.004**	1.59	0.95
Social/emotional reciprocity	56.50(43.00;79.25)	25.00(23.00;37.25)	6.00	0.746	**0.001**	2.04	1.00
Atypical language	44.00(36.00;53.75)	39.00(36.00;39.00)	31.00	0.328	0.165	0.92	0.61
Stereotypy	34.00(30.00;45.00)	31.00(30.00;32.00)	43.50	0.114	0.631	0.80	0.51
Behavioural rigidity	48.00(35.75;66.75)	25.00(25.00; 33.00)	10.50	0.682	**0.002**	1.66	0.97
Sensory sensitivity	51.00(36.50;59.00)	37.00(35.00;37.00)	19.00	0.543	**0.019**	1.59	0.95
Attention	62.00(44.00;72.75)	32.00(25.00;55.00)	21.00	0.491	**0.029**	1.05	0.71

Analysing the general level of intelligence on the basis of the results obtained we can conclude that experimental group patients with a lesion located in the cerebellum obtained significantly lower scores than healthy children on the IDS general intelligence screening index and the Categories and Matrices subscales. In terms of executive functions patients with cerebellar damage scored significantly lower on the Divided attention and Drawing routes IDS-2 subscales compared to the control group. There were not significant differences in Fluency subtest, and Colours subtest as well as in General Scores of EF between groups.

The results of the Conners 3 Questionnaire indicate that there were no differences between the cerebellar patients and control groups in the factors analysed in the questionnaire.

The analysis of the ASRS questionnaire results showed that the patients from the cerebellar lesion group obtained statistically significantly higher results than the control group in the following factors: social/communication, unusual behaviours, self-regulation, peer socialisation, adult socialisation, social/emotional reciprocity, behavioural rigidity, sensory sensitivity and attention (according to the assumptions of this test, a higher score in a given factor means a greater intensity of difficulties in a given area).

However, the study groups are small and in order to assess the probabilities of detecting a significant effect assuming the group size was larger, a post -hoc power analysis based on Cohen’s *d* coefficient effect size estimates (G*Power 3.1.9.4 software) was also conducted. Power analysis enables researchers to determine sufficient sample size for achieving adequate statistical power for given effect size. Statistical power depends on three parameters: significance level (α level), effect size, and sample size. Given an effect size value and a fixed α level, recruiting more participants in a study increases statistical power and the accuracy of the result. Power analysis was used in our study to estimate the minimum sample size required for a given population effect size to be significant, thus allowing to evaluate whether the effect should be expected if sample size was slightly larger. The results are presented below.

On the Animal Colours EF subscale of IDS-2 scale, the patient group scored lower on average (med_p_ = 10.50; Q_1_ = 4.75, Q_3_ = 11.00) compared to the control group (med_k_ = 10.00; Q_1_ = 9.00, Q_3_ = 11.50), but the difference was not statistically significant (*U* = 41.50, *p* = 0.529). The effect size was medium (*r* = 0.14, *d* = 0.67; cf. Ellis, 2010), the probability of detecting an effect was 1 – β = 0.41. The results would be statistically significant if the sample size would be increased by 50% (np = 15, nk = 15).

A similar effect was observed for the EF total score, the patient group scored lower on average (med_p_ = 7.00; Q_1_ = 3.25, Q_3_ = 9.75) compared to the control group (med_k_ = 9.50; Q_1_ = 7.75, Q_3_ = 13.00), but the difference was not statistically significant (U = 25.50, *p* = 0.063). The effect size was large (*r* = 0.41, *d* = 0.94; cf. Ellis, 2010), the probability of detecting an effect was 1 – β = 0.63, increasing the sample size by 50% (np = 15 and nk = 15) would result in statistical significance.

Increasing the group size by 50% could also help detect statistical significance of the difference between the experimental and control group in the following ASRS subscales: atypical language (*r* = 0.32, *d* = 0.92, probability of detecting an effect was 1 – β = 0.61) and stereotypes (*r* = 0.11, *d* = 0.80, probability of detecting an effect was 1 – β = 0.51).

In the case of the Conners 3 questionnaire, *post- hoc* power analysis based on Cohen’s *d* coefficient demonstrated that on the peer relationship subscale, the patient group scored higher on average (med_p_ = 45.50; Q_1_ = 43.00, Q_3_ = 55.25) compared to the control group (med_k_ = 42.50; Q_1_ = 40.75, Q_3_ = 47.75), but the difference was not statistically significant (*U* = 30.00, *p* = 0.143). The effect size was medium (*r* = 0.34, *d* = 0.59; cf. Ellis, 2010), the probability of detecting an effect was 1 – β = 0.34. Increasing in sample size by 70% (np = 16 and nk = 16) would result in statistical significance.

The above analyses for the other subscales examined, where no significant differences were detected between the study groups, showed that the effect sizes *d* were either close to or lower than 0.40, indicating that it would be necessary to considerably increase the group sizes by 300% (n_p_ >40 and n_k_ >40) or more for the effects to show up as significant. Therefore, it can be assumed that statistically significant differences are unlikely to show up in these subscales in a clinical study.

Spearmann correlation analysis was used to assess the association between motor impairment in the form of cerebellar ataxia severity measured with ICARS Scale and cognitive-emotional variables in the group of cerebellar patients. The mean score in ICARS Total is 10.3, the mean score of ICARS posture is 6, the mean score of ICARS limb functions is 4.1, the mean score of ICARS dysarthria is 1.3, the mean score of ICARS oculomotor functions is 0.4.

The results indicate the presence of a negative correlation between the ICARS posture score and the Matrices IDS subtest (*p* = 0.021, *R* = –0.713). The ICARS limb score also correlates negatively with Matrices IDS subscale(*p* = 0.022, *R* = –0.707). The ICARS dysarthria score correlates negatively with the divided attention variable (*p* = 0.001, *R* = –0.872), Categories IDS Score (*p* = 0.013, *R* = –0.746) and the overall executive function score (*p* = 0.013, *R* = –0.749). The ICARS total ataxia index correlates negatively with the Matrices IDS subscale (*p* = 0.018, *R* = –0.724).

Spearmann correlation analysis was performed in order to assess the relationship between emotional-social difficulties and the efficiency of executive functions in the studied group of children with a lesion in the cerebellum region. The results obtained indicate the presence of a negative correlation between the EF listing words subtest and the ASRS peer relationship sociality factor (*p* = 0.039, *R* = –0.656). The EF divided attention subtest correlates statistically significantly negatively with the ASRS peer relationship sociality factor (*p* = 0.044, *R* = –0.645). The EF drawing routes subtest correlates statistically significantly negatively with the Conners 3 Disobedience/aggression factor (*p* = 0.001, *R* = –0.884), ASRS self- regulation factor (*p* = 0.025, *R* = –0.695), ASRS Adult socialisation (*p* = 0.022, *R* = –0.708), ASRS behavioural rigidity factor (*p* = 0.004, *R* = –0.652). The overall executive function score correlates negatively with the Conners Inattention factor (*p* = 0.026, *R* = –0.695), ASRS social relationship/communication (*p* = 0.038, *R* = –0.660), ASRS Emotional reciprocity (*p* = 0.025, *R* = –0.697) and ASRS Attention (*p* = 0.015, *R* = –0.736).

Spearmann correlation analysis was performed in order to assess the relationship between age of the cerebellar patients and their executive functions performance. The obtained results did not confirmed the statistically significant corelations between these statistical variables (all *p*-values > 0.05).

We additionally wanted to check if vermis damage may influence the worse functioning in executive and emotional conditions. We divided the group of cerebellar patients into two groups (with and without vermis damage) and performed the Mann–Whitney *U* analysis. The patients with additionally vermis damage obtained significantly lower results than patients with mainly cerebellar hemispheric lesions in divided attention executive subscale (*p* = 0.030) and executive functions total score (p = 0.010). There is no difference in ASRS general result (*p* = 0.054).

## Discussion

The aim of our study was to describe the cognitive (in terms of executive functions), emotional and social functioning of children with lesions located in the cerebellum. Our study was preliminary, based on a very small group of subjects, but its aim was to determine the existence of the very phenomenon of impaired executive functioning in children with cerebellar damage, to delineate and specify further directions of research in this topic and to establish neuropsychological tools, sensitive enough to detect even subtle changes in the sphere of executive and behavioural functioning of these children.

Despite such a small study group, it was possible to obtain preliminary data on the presence of statistically significant differences in selected indicators of cognitive-social-emotional functioning between children with a lesion located in the cerebellum and healthy children. By analysing the power of the test in a *post hoc* model based on effect size estimates with Cohen’s *d* coefficient, it was possible to determine those variables at which, with a potential increase in group size, there was a higher probability of detecting an effect.

With regard to the executive function profile itself, preliminary analyses showed statistically significant differences between the cerebellar patients and the children in the control group in planning ability and divided attention. This result brings to light the role of the cerebellum as a structure that is part of neuronal networks supporting executive functions in children.

Based on preliminary observations, it may be assumed that children with cerebellar damage may present impaired efficiency of selected executive functions. Particular difficulties may be caused by tasks requiring divided attention, locating its resources simultaneously on two competing cognitive activities. In addition, these children may find it difficult to perform tasks requiring planning, organisation of complex activities, inhibition of impulsive, automatic reactions, which was revealed in the IDS-2 drawing routes subtest, in which the task was to draw a route in such a way that each section is marked, and there is no possibility of returning to already marked ones. This requires pre-planning the route, and a characteristic of the cerebellar children tested was a revealed tendency to act without prior thought, impulsively without thinking. A difficulty with cause-and-effect thinking was evident.

The results described above confirm observations of executive dysfunction made in other studies involving children after cerebellar tumour surgery. Starowicz-Filip et al. (2017) in a study of visuospatial functions in children after surgery for benign cerebellar tumour, indicated executive difficulties in visuospatial organisation and planning, evident in the task of copying the Rey-Osterrieth Complex Figure. Planning difficulties were also found in this group of children in the area of learning new auditory-verbal material in the Rey 15 Item Test (Starowicz-Filip et al., 2020). The weaker growth of new memorised items in the learning process was secondarily attributed to a defective organisation and semantic association strategy of successive words of the test. Cerebellar patients formed significantly fewer semantic clusters, as well as did not use such mnemonics as categorisation, which could have helped them acquire the presented word list more effectively. Karatekin et al. (2000) studied four children after surgery for a tumour located in the cerebellar hemispheres using the Wisconsin Card Sorting Test and compared their results with those of 6 children after tumour or temporal lobe cyst removal. The cerebellar patients showed difficulty in using feedback in discovering and generating further routines, after discovering and completing the first category fairly quickly. The temporal patients presented a different pattern of dysfunction, involving difficulty in initially formulating the first rule of the test and sustaining attention. Koustenis et al. (2016), in a study of 42 children after cerebellar tumour surgery (17 patients after surgery for a lower grade tumour and 25 patients with a highly malignant tumour of this structure), found difficulties in executive functions such as cause-and-effect thinking, cognitive control and flexibility. In addition, these researchers (Koustenis et al., 2016) reported poorer performance on the Tower of London test, based on the analysis of planning ability and causal thinking, but only in children after surgery for a highly malignant tumour who had also undergone radio- and chemotherapy treatment. On the other hand, Riva and Giorgi (2000) neuropsychologically examined children 5–6 weeks after cerebellar tumour surgery and demonstrated that, regardless of the level of tumour malignancy, these patients presented significantly elevated perseverative errors on the Wisconsin Card Sorting Test, at least two standard deviations from the norm. Vaquero et al. (2008) in a longitudinal study showed that deficits in planning were still present in patients after surgery for a high-grade tumour six years after surgery. In children with cerebellar tumours of lower malignancy, these problems regressed after about three years, unless the lesion involved the dentate nucleus, in which case the dysfunctions appeared to be permanent. Kipping et al. (2018) provided evidence that planning ability during childhood could partially be achieved through the engagement of the lateral cerebellum.

The difficulty in divided attention that emerged in our study could be linked to the increasingly popular hypothesis regarding the role of the cerebellum in the so-called multitasking. The cerebellum plays a role in the automation of activities, not only motor but also cognitive. Effectively performing two tasks simultaneously requires at least one of them to be automated, so that attentional resources can be more successfully allocated to the task that requires more focus (see Bellebaum and Daum, 2007).

The presence of executive dysfunction in children with cerebellar damage may be explained neurofunctionally, according to the connectionist model, by numerous connections between the cerebellum and the prefrontal brain region (Schmahmann and Pandya, 1997) directly responsible for the regulation of executive functions (Robbins, 1996) (fronto-ponto-cerebellar pathway and cerebello-parietal pathway). Neuroimaging and neuropsychological studies, also using animal models, emphasise the role of the cerebellum as a modulator of prefrontal cortex function, such as planning, control of inhibition, divided attention, fluency (Middleton and Strick, 2001). Myelination of the aforementioned connections between the cerebellum and the PFC takes place from childhood to adolescence. Planning, goal-setting, and cognitive flexibility mature relatively late into middle adolescence, whereas attention, self-regulation, and control mechanisms emerge as early as early childhood (Jacobs and Anderson, 2002). Therefore, damage to the area of developing cerebellocortical connections makes patients vulnerable to the emergence of the executive dysfunctions mentioned above.

However, Beuriat et al. (2022) stated that the importance of the role of the cerebellum in executive functions is particularly crucial in newborns and young children when the EF, motor control and perception are more closely interrelated. The cerebellum due to its important role in motor control and sequencing makes executive functions more reliant on these computational properties that compute spatial distance, motor intent, and assist in the execution of sequences of behaviour.

In our study the observed effect of executive dysfunctions was worse in those patients with additional vermis lesion. Many studies underline that vermal damage is strongly associated with emotional and behavioural disturbances as well as may worsen cognitive functioning (Steinlin et al., 2003).

Referring back to the preliminary profile of executive dysfunctions of children after cerebellar tumour surgery that we obtained, the lack of differences in a profile variable such as verbal fluency between children with cerebellar damage and healthy children certainly warrants further exploration, as previous studies show that cerebellar lesions, especially of its right hemisphere, are strongly correlated with verbal fluency disorders (Fiez et al., 1992; Arasanz et al., 2012). It should be noted that studies to date mainly include the adult patient population. Perhaps the reason for this discrepancy should be attributed to the fact that in our small cerebellar group, three children were younger (up to 9 years of age) and for this age group testing based solely on semantic fluency is applicable, which, according to research, is less correlated with frontal lobe performance, in contrast to letter fluency, which is seen as a much better measure of executive function (Henry and Crawford, 2004). Interestingly, the Stroop effect in the IDS-2 animal colours subtest was also not found in cerebellar patients. One might conclude that these children would have no difficulty in inhibiting automatic misreaction, but at the same time the IDS-2 drawing routes test revealed their high impulsivity, lack of an action plan and difficulties in cause-and-effect thinking. The explanation of this emerging tendency needs verification but it may be due to the conditions of the task. In the IDS-2 animal colours task, the child has a clear and single goal - to control the naming of the colours of the animals seen, in order to “recall from memory” the correct colour of the animal, whereas in the IDS-2 drawing routes subtest this element of control and pre-planning, is somewhat hidden and obscured by another requirement of the task—to find a solution quickly. Cerebellar patients apparently find it difficult to multitask here—to keep in mind the goal and at the same time to think about not repeating the route once taken.

Regarding the analysis of cognitive functioning, children with lesions located in the cerebellum presented a significantly lower level of intelligence measured by screening with the IDS-2 test in comparison to healthy children from the control group. At the same time, these results were still within the range of average intelligence (median 98.50, lower quartile 87.25). Our results are consistent with the observations of other authors (Mabbott et al., 2008; Koustenis et al., 2016) who report that in children after surgery for benign cerebellar tumours (without radio- or chemotherapy) the general level of intelligence usually does not decrease, or if it does, it is still at the level of average intelligence, which may mask deficits in executive functions.

The analysis of emotional-behavioural changes in children with lesions located in the cerebellum region brought more conclusive results in comparison with the results in the executive functions, even considering such a small study group. The results obtained based on the ASRS questionnaire showed that cerebellar patients presented significantly higher intensity of selected behaviours similar to that observed in the autism spectrum in comparison with the group of neurologically healthy children. According to the parents’ description, cerebellar patients are characterised by deterioration of communication skills in establishing new social relationships, both with adults and peers, greater difficulty in self-regulation of emotions (greater impulsivity, irritability, and explosiveness), difficulties in the so-called social reciprocity, which consists, among other things, in analysing and understanding other people’s emotional states, the level of empathic abilities, increased rigidity of behaviour and difficulty in abandoning once established patterns of behaviour, changing plans, weaker ability to concentrate attention. The ASRS questionnaire may prove to be a much more sensitive tool for detecting emotional and behavioural changes than the Conners 3 questionnaire. The parents of the children in the study did not mark traits of hyperactivity/impulsivity, difficulties with learning, attention, executive functions, although in the interviews about their children’s functioning they clearly highlighted these very difficulties. This discrepancy between data obtained from parents during the initial interview about their child’s functioning and the results of the Conners questionnaire will require further in-depth exploration. In the aforementioned study by Karatekin et al. (2000), which reported executive function deficits in children following cerebellar tumour surgery, their parents did not cite executive dysfunction as a major problem for these children in their initial interviews, but instead reported precisely emotional difficulties: low frustration tolerance, increased irritability and emotional hypersensitivity. Other studies also confirm that children after cerebellar tumour surgery both immediately after (Levisohn et al., 2000; Riva and Giorgi, 2000) and in a more distant perspective (Steinlin et al., 2003) present emotional lability, shallow affect, irritability, impulsivity, heightened anxiety and difficulties in emotion regulation. This constellation of disorders together with cognitive difficulties forms the cerebellar cognitive affective syndrome. The emotional component may be dominated by kind of “autistic” behaviours, which requires further exploration, however, the results of research highlighting the presence of structural anomalies in the structure of the cerebellum in children with autism are not without significance (Townsend et al., 2001).

However, it is crucial to mention that deterioration of communication skills in establishing new social relationships both with adults and peers, observed by the parents in cerebellar children might be the long term effect of the earlier cerebellar mutism syndrome or might be a manifestation of slight aphasia, which is sometimes detected in the cerebellar patients (Catsman-Berrevoets and Patay, 2018). This problem requires the further exploration.

Considering the impact of emotional difficulties on cognitive functioning, in our preliminary study we obtained a significant association between behavioural difficulties of an autistic behavioural nature, such as difficulties in social reciprocity, difficulties in adult or peer relations and poorer executive functioning. However, these relationships are not obvious and require further exploration. Rather, associations with variables examined by the Conners questionnaire such as impulsivity, irritability, and hyperactive traits were expected.

An important variable that requires further control in the context of our study is the presence of motor disorders of the cerebellar ataxia type, which may secondarily modify the level of performance on a number of tasks originally dedicated to the assessment of cognitive functions. Some of the correlations we obtained between particular dimensions of ataxia and cognitive variables such as inference and categorisation measured with the matrices test, which by definition does not require motor skills on the part of the child, are difficult to explain and are most likely random in nature. At the same time, the association of dysarthria with poorer performance in the divided attention test is logical, since rapid naming of animals requires an efficient articulatory function of speech.

### Limitations

Our study has some serious limitations that warrant mention. The most important are the small study sample and insufficient control of motor disorders. Moreover in some single cases (case no 9) we should have consider longer time of between neuropsychological exam and surgery to avoid the risk of analyzing the cognitive functions in the acute state after surgery.

## Conclusion

Our preliminary results suggest the possible presence of executive dysfunction in children operated for cerebellar tumours.

The executive functions most likely to be particularly sensitive to cerebellar lesions are divided attention, planning ability and impulsivity control.

Our preliminary results suggest the possible presence of emotional-behavioural changes in children with cerebellar damage, which are the most similar to some behaviours observed in autism spectrum disorders.

With regard to the diagnostic tools used, it seems that the IDS-2 battery of tests for the assessment of executive functions, as well as the ASRS questionnaire may prove to be sufficiently sensitive and accurate tools for the description of the part of the cognitive and emotional functioning of the children with cerebellar lesion. At the same time, the use of the Conners 3 questionnaire to assess the features of hyperactivity, irritability, impulsivity, executive function disorders proved to be problematic, and this tool may not be sensitive enough, in the context of discrepancies between the results obtained with its use and the reports from parents obtained in preliminary interviews.

The diagnostic awareness of possible cognitive-emotional difficulties in children with cerebellar damage will certainly contribute to a more accurate and individualised selection of neuropsychological rehabilitation programmes for patients, enabling a faster return to efficient school and social functioning.

## Data availability statement

The raw data supporting the conclusions of this article will be made available by the authors, without undue reservation.

## Ethics statement

The studies involving human participants were reviewed and approved by Bioethics Committee of Jagiellonian University Medical College. Written informed consent to participate in this study was provided by the participants’ legal guardian/next of kin.

## Author contributions

BB-K: text editing. TY: supervising the manuscript and the course of the research and checking the text. SK, OM, and ŁK: taking care of neurological and neurosurgical parts of our research and qualifications of the patients according to their neurological status. AC: supervising the manuscript and the course of the research. All authors contributed to the article and approved the submitted version.
